# Functional expression of the eukaryotic proton pump rhodopsin *Om*R2 in *Escherichia coli* and its photochemical characterization

**DOI:** 10.1038/s41598-021-94181-w

**Published:** 2021-07-20

**Authors:** Masuzu Kikuchi, Keiichi Kojima, Shin Nakao, Susumu Yoshizawa, Shiho Kawanishi, Atsushi Shibukawa, Takashi Kikukawa, Yuki Sudo

**Affiliations:** 1 Division of Pharmaceutical Sciences, Okayama University, Okayama, 700-8530 Japan; 2 Graduate School of Medicine, Dentistry and Pharmaceutical Sciences, Okayama University, Okayama, 700-8530 Japan; 3 Atmosphere and Ocean Research Institute, The University of Tokyo, Chiba, 277-8564 Japan; 4 Faculty of Advanced Life Science, Hokkaido University, Sapporo, 060-0810 Japan; 5 Global Station for Soft Matter, GI-CoRE, Hokkaido University, Sapporo, 001-0021 Japan

**Keywords:** Biochemistry, Biophysics, Chemistry

## Abstract

Microbial rhodopsins are photoswitchable seven-transmembrane proteins that are widely distributed in three domains of life, archaea, bacteria and eukarya. Rhodopsins allow the transport of protons outwardly across the membrane and are indispensable for light-energy conversion in microorganisms. Archaeal and bacterial proton pump rhodopsins have been characterized using an *Escherichia coli* expression system because that enables the rapid production of large amounts of recombinant proteins, whereas no success has been reported for eukaryotic rhodopsins. Here, we report a phylogenetically distinct eukaryotic rhodopsin from the dinoflagellate *Oxyrrhis marina* (*O. marina* rhodopsin-2, *Om*R2) that can be expressed in *E. coli* cells. *E. coli* cells harboring the *Om*R2 gene showed an outward proton-pumping activity, indicating its functional expression. Spectroscopic characterization of the purified *Om*R2 protein revealed several features as follows: (1) an absorption maximum at 533 nm with all-*trans* retinal chromophore, (2) the possession of the deprotonated counterion (p*K*_a_ = 3.0) of the protonated Schiff base and (3) a rapid photocycle through several distinct photointermediates. Those features are similar to those of known eukaryotic proton pump rhodopsins. Our successful characterization of *Om*R2 expressed in *E. coli* cells could build a basis for understanding and utilizing eukaryotic rhodopsins.

## Introduction

To capture sunlight, organisms use a variety of photoreceptive proteins that are responsible for light-energy conversion and light-signal transduction in nature. Photoreceptive membrane proteins called microbial rhodopsins form large phylogenetic clusters in three domains of life, archaea, bacteria and eukarya^1,2^. Microbial rhodopsins consist of seven-transmembrane α-helices covalently linked to the chromophore all-*trans* retinal, a derivative of vitamin-A^2,3^. The chromophore retinal covalently binds to a conserved Lys residue located in the 7th helix of the rhodopsin apoprotein through a protonated Schiff base linkage^3^. After photoisomerization of the retinal from an all-*trans* to a 13-*cis* configuration, microbial rhodopsins undergo a series of cyclic reactions called a photocycle in which several spectrally distinct photointermediates are sequentially formed and the initial state is recovered along with the conformational changes^3^. During each photocycle, rhodopsins exhibit their biological functions such as ion transport and photosensing^1–3^. For instance, outward proton pumps produce the molecular currency adenosine triphosphate (ATP) through the formation of a proton gradient across the cell membrane like photosynthesis, indicating their physiological significance in microorganisms^4^.

Archaeal and bacterial rhodopsins have been the basis for research of microbial rhodopsins. Historically, bacteriorhodopsin (BR) was first rhodopsin discovered from the halophilic archaea *Halobacterium salinarum* as an outward proton pump in 1971^5^. After that, halorhodopsin and sensory rhodopsin I and II were identified from the halophilic archaea as an inward chloride pump and a phototaxis sensor, respectively^1^. Many other archaeal rhodopsins have also been extensively characterized photochemically and their ion transport and signal transduction mechanisms have been established at the atomic level with a high temporal resolution^3,6^. In 2000, a bacterial outward proton pump rhodopsin, proteorhodopsin (PR), was identified from the marine γ-Proteobacteria, which opened a new era of genomic exploration of unknown microbial rhodopsins^7^. Since then, numerous bacterial rhodopsins, such as a thermally stable outward proton pump rhodopsin, thermophilic rhodopsin (TR), were identified from the extremely thermophilic bacterium *Thermus thermophilus*. An outward sodium pump rhodopsin, Krokinobacter rhodopsin 2 (KR2) from *Krokinobacter eikastus*, and a transcriptional regulator, Anabaena sensory rhodopsin (ASR), were unveiled by genomic analysis and have been extensively characterized as have archaeal rhodopsins^7–12^. To photochemically characterize rhodopsins in detail, large amounts of proteins are required. For rhodopsins from *H. salinarum*, abundant proteins can be obtained from the native and mutant strains of *H. salinarum*, which has accelerated their research. However, it has been difficult in general to obtain native proteins of many microbial rhodopsins from native organisms due to their low expression levels and the difficulty of cultivating cells that express them. In 1997, the functional expression of *Natronomonas pharaonis* phoborhodopsin (*p*pR) in *Escherichia coli* was achieved by Shimono et al.^13^, by which the acquisition of large amounts of proteins and the efficient production of many mutants can be performed efficiently. Since then, the *E. coli* expression system has been widely and successfully used for several types of microbial rhodopsins including the rhodopsins mentioned above^9,14–16^,*E. coli*-based expression system is the foundation for the molecular analysis of microbial rhodopsins.

**Figure 1:**
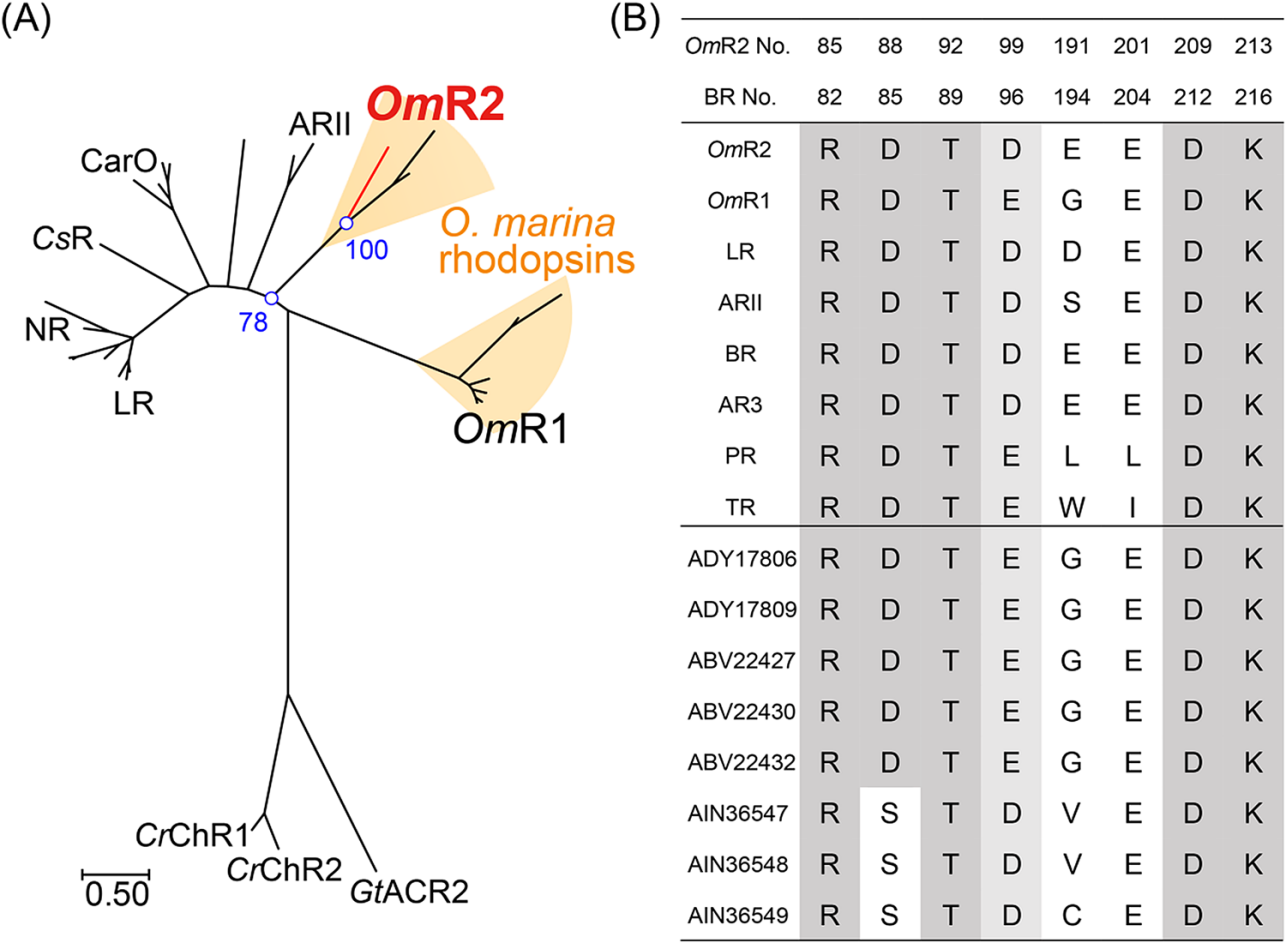
Characteristics of the eukaryotic rhodopsin *Om*R2. (**A**) Phylogenetic tree of microbial rhodopsins from eukaryotes (see “Methods” for detail). The scale bar indicates the number of substitutions per site. The numbers (i.e., 78 and 100) represent the bootstrap probabilities. (**B**) List of representative amino acid residues responsible for the function in *Om*R2 and the well-characterized outward proton pump rhodopsins, *Om*R1, LR, ARII, BR, AR3, PR, TR and *O. marina* rhodopsins (Genbank accession number: ADY17806, ADY17809, ABV22427, ABV22430, ABV22432, AIN36547, AIN36548, AIN36549). Abbreviations of the rhodopsins are as follows: Acetabularia rhodopsin II, ARII; archaerhodopsin-3, AR3; bacteriorhodopsin, BR; *Chlamydomonas reinhardtii* channelrhodopsin-1, *Cr*ChR1; *Chlamydomonas reinhardtii* channelrhodopsin-2, *Cr*ChR2; *Coccomyxa subellipsoidea* rhodopsin, *Cs*R; *Fusarium fujikuroi* rhodopsin, CarO; *Guillardia theta* anion channelrhodopsin-2, *Gt*ACR2; *Leptosphaeria maculans* rhodopsin, LR; *Neurospora crassa* rhodopsin, NR; *Oxyrrhis marina* rhodopsin-1, *Om*R1; *Oxyrrhis marina* rhodopsin-2, *Om*R2; proteorhodopsin, PR; thermophilic rhodopsin, TR.

Recently, advances in genomic analysis have revealed the presence of numerous microbial rhodopsins in eukaryotes (Fig. 1A). *Neurospora crassa* rhodopsin (NR) was identified from the filamentous fungus, *Neurospora crassa*, in 1999 although its molecular function was unclear^17^. After that, eukaryotic proton pump rhodopsins such as Leptosphaeria rhodopsin (LR) from *Leptosphaeria maculans*, Acetabularia rhodopsin I and II (ARI and ARII) from *Acetabularia acetabulum*, *Oxyrrhis marina* rhodopsin-1 (OR1 or *Om*R1) from the dinoflagellate, cation channel rhodopsins such as channelrhodopsin-1 and -2 from the alga *Chlamydomonas reinhardtii* (*Cr*ChR1 and *Cr*ChR2) and anion channelrhodopsins such as anion channelrhodopsin-1 and -2 from the alga *Guillardia theta* (*Gt*ACR1 and *Gt*ACR2), have also been identified and characterized^18–20^. In addition, eukaryotic ion transporting rhodopsins have attracted attention as a molecular tool for optogenetics^21^. While cation channelrhodopsins are used to induce neural activation, outward proton pump rhodopsins and anion channelrhodopsins are used to induce neural silencing^21,22^. Therefore, the molecular characterization of eukaryotic ion transporting rhodopsins should provide useful information for their modification and development as new optogenetics tools.

Although eukaryotic rhodopsins can be characterized by heterologous expression systems using yeast cells, *Xenopus* oocytes, mammalian cells, insect cells and cell-free systems^23–25^, it is generally difficult to achieve their functional expression in *E. coli* cells. Nonetheless, we previously succeeded in expressing *Cr*ChR1 in *E. coli* cells by truncating the N- and C-termini of the proteins, although the truncated mutants showed constitutive activities that are different from the wild-type proteins^26^. We also succeeded in the functional expression and mutational analysis of *Gt*ACR2 in *E. coli* cells, which could provide a characteristic mutant for new optogenetics tools^27–29^. However, we were not able to purify the photoactive protein of *Gt*ACR2 from *E. coli* cells probably due to its denaturation during the solubilization step in detergent micelles. One research group fused the Mistic domain, a membrane-associated protein from *Bacillus subtilis*, into the N- and C-termini of ARI and *Cr*ChR1, to allow their functional expression in *E. coli* cells and successfully characterized the photochemical properties of the purified proteins^30^. However, the yields of the purified proteins were 0.12 mg for Mistic-fused ARI and 0.04 mg for Mistic-fused *Cr*ChR1 per liter of culture medium, which is more than tenfold lower than those of bacterial rhodopsins (2 and 5 mg for TR and *Salinibacter ruber* sensory rhodopsin I, respectively)^9,15^.

While organisms usually have several rhodopsin genes in their genome, the eukaryotic dinoflagellate *O. marina* uniquely shows more than 10 putative rhodopsin genes (Fig. 1B)^31–33^. *O. marina* is a heterotrophic dinoflagellate that is widely distributed on earth^34^. Noteworthy, *O. marina* shows several important characteristics as follows: (1) it can be isolated from the environment and easily cultured in medium, (2) genetic approaches are available, and (3) it is inexpensive to obtain, maintain and is practical to use. Therefore, *O. marina* has been widely used as a model for dinoflagellates for over 100 years in various scientific fields including phylogeny, biogeography and ecology^35^. By employing those characteristics, it has been reported that putative rhodopsin genes can be expressed as transcripts and proteins in *O. marina*, which suggests that those genes encode functional proteins^31,32^. Hartz et al. reported that *O. marina* can orient to light based on rhodopsins and may use that photosensory response to detect algal prey based on chlorophyll autofluorescence^36^. Based on that background, we assume that *O. marina* rhodopsins (*Om*Rs) would be good candidates for the functional expression and analysis of eukaryotic rhodopsins. So far, one *Om*R, *Om*R1 (Genbank accession number: ABV22426) has been characterized by a heterologous expression system in yeast, but not by the *E. coli* cell expression system^37^. Thus, for expression in *E. coli* cells, we focused on the other gene named *O. marina* rhodopsin-2 (*Om*R2) (Genbank accession number: AIN36546). *Om*R2 contains several amino acids that are responsible for its outward proton pump functions such as Asp88, Thr92, Asp99 and Asp209, which correspond to Asp139, Thr143, Asp150 and Asp266 in LR, respectively (Fig. 1B and Fig. S1). Thus, *Om*R2 should work as an outward proton pump. It is noteworthy that *Om*R2 is phylogenetically distinct from *Om*R1 and other eukaryotic rhodopsins (Fig. 1A), where the amino acid identities and similarities of *Om*R2 with LR (15.8% identity, 32.1% similarity) and *Om*R1 (17.8% identity, 39.4% similarity) are relatively lower than those between other characterized eukaryotic rhodopsins, suggesting its phylogenetically distinct feature.

In this study, we characterize the function and molecular properties of *Om*R2 as a new model for eukaryotic rhodopsins. We show that *Om*R2 can functionally work in *E. coli* cells as a recombinant protein with a light-driven outward proton pump activity, which is confirmed to be consistent with the electrophysiological results obtained with the mammalian expression system. By taking advantage of the *E. coli* cell expression system, we obtained highly purified photoactive proteins and successfully performed spectroscopic analyses. The results indicate that *Om*R2 has similar photochemical properties to the well-characterized eukaryotic proton pump rhodopsins, which suggests that *Om*R2 can be a model for eukaryotic proton pump rhodopsins. Thus, the successful expression, purification and characterization of *Om*R2 in *E. coli* cells could build a basis towards understanding the molecular mechanism of eukaryotic rhodopsins.

## Results and discussion

### Absorption spectrum and electrophysiological experiments of *Om*R2 in mammalian cells

**Figure 2:**
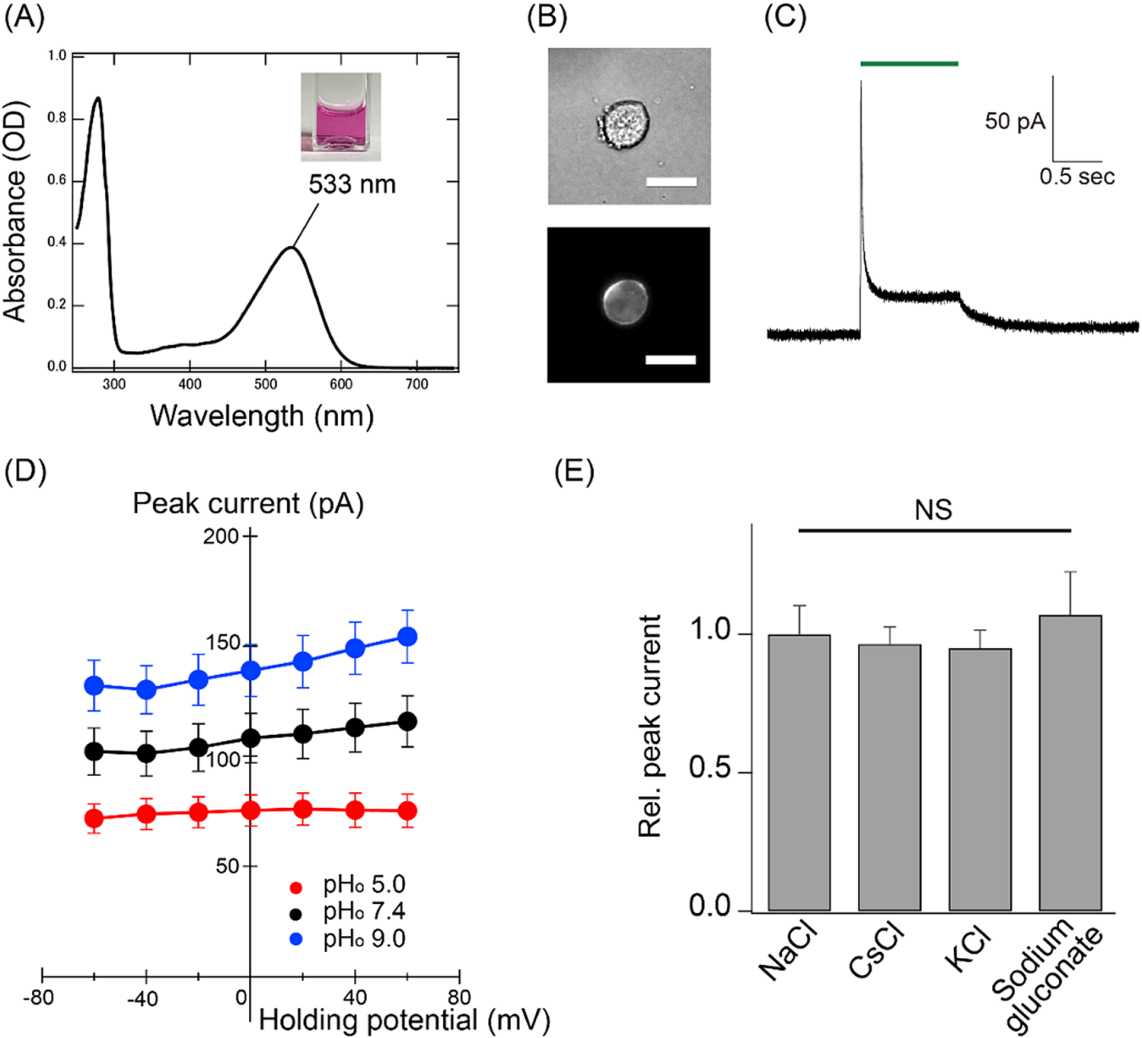
Absorption spectrum and electrophysiological experiments of *Om*R2 in mammalian cells. (**A**) Absorption spectrum of *Om*R2 purified from HEK293 cells in Buffer A containing 50 mM Tris–HCl (pH 7.0), 1 M NaCl and 0.05% (w/v) DDM. The inset photograph represents the color of purified *Om*R2. (**B**) Phase-contrast image (upper panel) and fluorescence image (lower panel) of ND7/23 cells with the expression plasmid of *Om*R2. The scale bars represent 30 µm. (**C**) Light-induced photocurrent signal at the membrane potential of 0 mV. The green bar indicates the period of illumination for 1.0 s. (**D**) Current–voltage relationship *(I–V* curve) of *Om*R2. The intracellular pH values were fixed at 7.3. Error bars indicate S.E. (n = 11–19 cells). (**E**) Comparison of relative peak currents at 0 mV with different extracellular medium conditions. There was no significant difference between the value with NaCl and the other values (P > 0.05; Dunnett’s test).

To investigate whether the *Om*R2 gene encodes a photosensitive protein, we first expressed and purified its recombinant protein using the HEK293 cell expression system, which has been utilized for the functional expression of several eukaryotic rhodopsins from microbes and animal rhodopsins^29,38^. The purified *Om*R2 protein in detergent DDM micelles was colored purple and its absorption spectrum showed an absorption peak at 533 nm (Fig. 2A), indicating that *Om*R2 works as a green light sensitive protein.

We then performed an electrophysiological study to investigate the function of *Om*R2. For expression in ND7/23 cells, the cDNA for *Om*R2 and EYFP were inserted downstream of the CMV promoter with the trafficking signal (TS) and endoplasmic reticulum export signal (ER) to enhance the membrane localization. The corresponding gene constructs have been utilized for functional expression in mammalian cells for various kinds of microbial rhodopsins such as sodium pump rhodopsins and halorhodopsin^39,40^. For transfected ND7/23 cells, the yellow fluorescence from EYFP was observed especially at the plasma membrane of the cells (Fig. 2B). This indicates the successful expression and localization of *Om*R2 in the plasma membrane. We then performed electrophysiological analysis to confirm the ion transport activity of *Om*R2. A positive photocurrent upon illumination was observed under conditions where the extracellular and intracellular pH were 7.4 and 7.3, respectively, and the holding membrane potential was 0 mV (Fig. 2C). We also measured the peak currents at membrane potentials from − 60 to 60 mV to obtain the current–voltage relationship (*I–V* curve) (Fig. 2D). The positive peaks were kept at all potentials, which suggests that *Om*R2 works as an outward cation pump or an inward anion pump. To further identify the substrate ion of *Om*R2, we measured the photocurrents under different extracellular ion compositions. It was first observed that the higher the extracellular pH was set, with an intracellular pH of 7.3, the higher the positive peaks were at all membrane potentials (Fig. 2D). Thus, the amplitudes of peak currents were sensitive to the extracellular pH values. On the other hand, when NaCl in the extracellular solution was replaced by CsCl, KCl or sodium gluconate, no significant change in the amplitudes was observed (Fig. 2E). These results indicate that *Om*R2 works as a light-driven outward proton pump. The peak photocurrent (~ 100 pA) was comparable to that of archaerhodopsin-3, AR3 (~ 100 pA)^41^ and a sodium pump rhodopsin KR2 (~ 100 pA)^40^. The successful expression and robust outward photocurrents of *Om*R2 suggest its applicability as a neural silencing tool for optogenetics similar to AR3 and KR2^22,40^.

### Functional expression of *Om*R2 in *E. coli* cells

**Figure 3:**
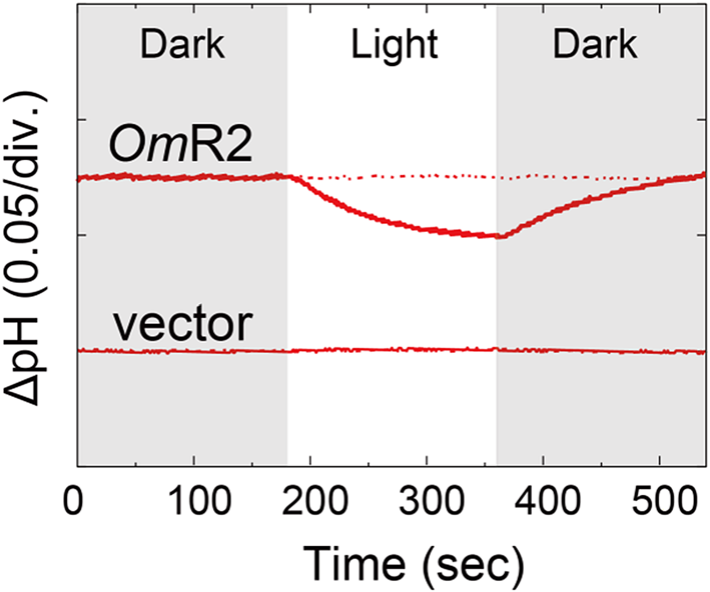
Outward proton pump activity of *Om*R2 in *E. coli* cells. Light-induced pH changes of solutions containing *E. coli* cells with the expression plasmid for *Om*R2 (upper panel) or the empty vector pET21a (lower panel) in the presence (red dashed lines) or absence (red solid lines) of the proton-selective ionophore, CCCP (10 µM). The initial pH ranged from 6.4 to 6.6. The white-filled region indicates the period of illumination.

So far, the *E. coli* cell expression system has been widely utilized for various archaeal and bacterial rhodopsins^9–13,39,41,42^. Since *Om*R2 is phylogenetically distinct from the other characterized eukaryotic rhodopsins, we sought to express the *Om*R2 recombinant protein using the *E. coli* cell expression system. We cultured *E. coli* BL21(DE3) cells harboring expression plasmids of *Om*R2. To prove that the *Om*R2 protein works as a photoactive protein, its light-dependent ion transport activity was observed as light-induced pH changes of a suspension of *E. coli* cells (Fig. 3). Illumination induced a pH decrease in the cell suspension, which would reflect the outward proton movement across the membrane while no pH decrease was observed in *E. coli* cells harboring the empty vector without the *Om*R2 gene (Fig. 3). The pH change disappeared in the presence of a proton-selective ionophore, CCCP, which works to collapse the proton motive force across the membrane. These results indicate that *Om*R2 has a light-dependent outward proton transport activity. In other words, *Om*R2 works as an outward proton pump in *E. coli* cells, which is consistent with the electrophysiological results for *Om*R2 expressed in ND7/23 cells (Fig. 2). Therefore, we concluded that the functional expression of *Om*R2 can be realized as a recombinant protein in *E. coli* cells. As far as we know, there has been no report that successfully obtained recombinant proteins of eukaryotic rhodopsins in *E. coli* cells, except for a few examples^26,27,30^.

To discuss what is the factor required for the successful expression of *Om*R2 in *E. coli* cells, we compared the amino acid sequences of *Om*R2 and other typical eukaryotic rhodopsins (Fig. 1). Among the characterized eukaryotic rhodopsins, NR is the phylogenetically closest to *Om*R2 but cannot be functionally expressed in *E. coli* cells in amounts sufficient for analysis^43^. It should be noted that the amino acid identity and similarity between them are 19.8 and 41.3%, respectively, and therefore it is difficult to identify which element is essential for the functional expression of *Om*R2 in *E. coli* cells from the comparison between them. In addition to *Om*R2, uncharacterized and phylogenetically distinct microbial rhodopsins have been continuously identified from various eukaryotes. From comprehensive comparisons of amino acid sequences among these possible molecules that can be functionally expressed in *E. coli* cells, it may be possible to identify which region in eukaryotic rhodopsins is responsible for the functional expression in *E. coli* cells. To prove this concept, we will identify other molecules that can be functionally expressed in *E. coli* cells from a comprehensive expression analysis of eukaryotic rhodopsins. The region(s) conserved among them will then be introduced into molecules that could not be expressed in *E. coli* cells, such as NR, LR and *Om*R1. That approach should lead to the identification of underlying element(s) in eukaryotic rhodopsins that allow the successful functional expression in *E. coli* cells.

In addition to the sequence information, it is known that post-translational modifications (PTMs), such as glycosylation and disulfide bond formation, play important roles in the functional and structural maintenance of membrane proteins while many types of PTMs are deficient in bacteria^44^. We speculate that eukaryotic rhodopsins can be expressed in mammalian cells partially due to PTMs. The PTMs of *Om*R2 are still unclear, and therefore, further investigation is required in the future.

### Purification and photochemical properties of *Om*R2

**Figure 4:**
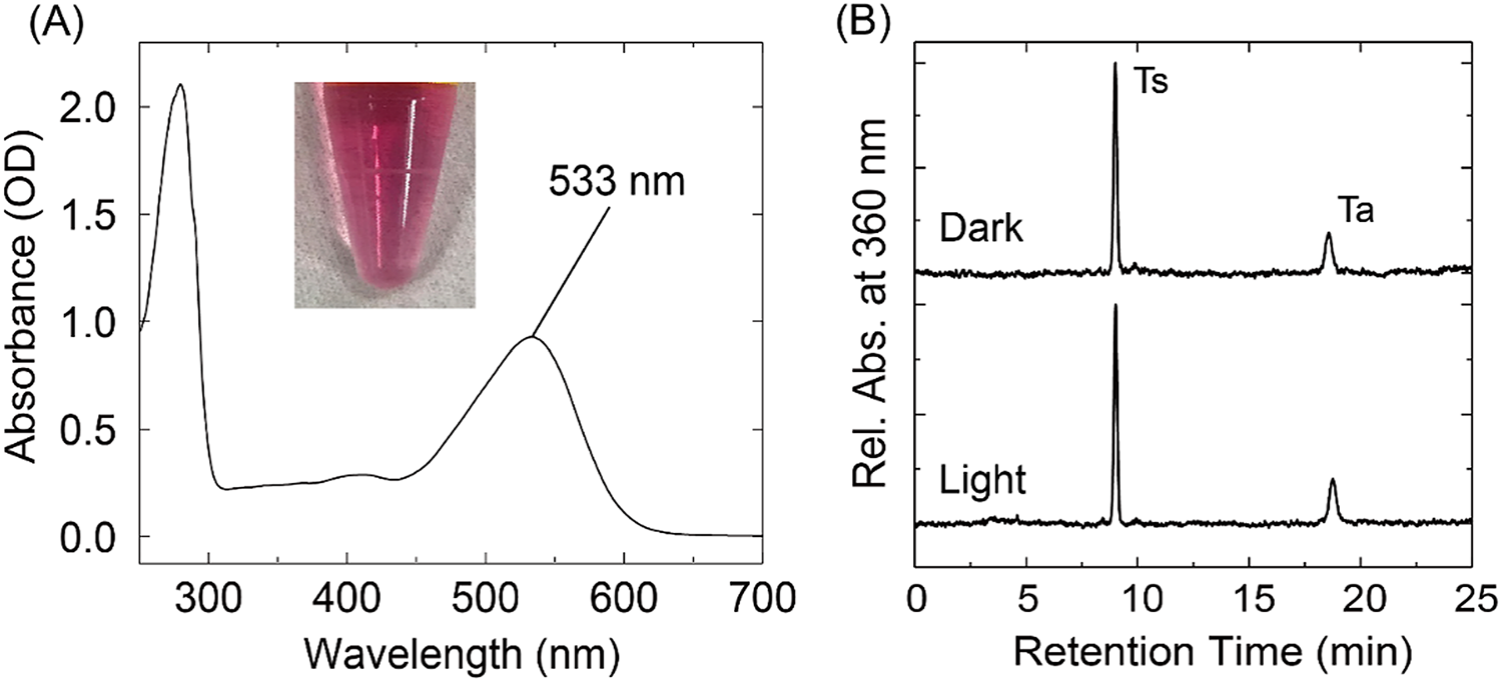
Absorption spectrum and retinal configuration of *Om*R2 expressed in *E. coli* cells*.* (**A**) Absorption spectrum of *Om*R2 purified from *E. coli* cells in Buffer A containing 50 mM Tris–HCl (pH 7.0), 1 M NaCl and 0.05% (w/v) DDM. The inset photograph represents the color of purified *Om*R2. (**B**) HPLC patterns of *Om*R2 in dark- and light-adapted states (upper and lower traces, respectively). Ts and Ta represent all-*trans*-15-*syn* and all-*trans*-15-*anti* retinal oximes, respectively.

As described in “Methods”, the *E. coli* cells were solubilized in DDM, after which the solubilized *Om*R2 proteins were purified by Ni-affinity column chromatography and had a purple color similar to the purified *Om*R2 expressed in HEK293 cells (Figs. 2A and 4A). The absorption spectrum of the purified *Om*R2 in *E. coli* cells showed an absorption peak at 533 nm (Fig. 4A), which is consistent with the result from the purified *Om*R2 in HEK293 cells (Fig. 2A). Thus, *Om*R2 obtained from *E. coli* cells forms a photoactive pigment in the detergent micelles without significant denaturation. As far as we know, this is the first demonstration where a eukaryotic rhodopsin was purified in detergent micelles using the *E. coli* cell expression system, except for the previous report of Mistic-fused ARI and *Cr*ChR1 proteins^30^. The yield of purified *Om*R2 protein was 1 mg per liter of culture medium, which is more than ninefold higher than the yields of Mistic-fused ARI (0.12 mg) and Mistic-fused *Cr*ChR1 (0.04 mg) and is comparable to the yield of TR (2 mg)^9^. The absorption of *Om*R2 at ~ 280 nm represents the absorption of aromatic residues such as Trp and Tyr. From the ratio of absorbance at 280 and 533 nm with the molecular coefficient of microbial rhodopsins (~ 50,000 cm^−1^ M^−1^), we roughly estimated the purity of the sample as 67.5%.

Using the purified proteins from *E. coli* cells, we performed the photochemical characterization of *Om*R2. We first performed HPLC analysis to determine the retinal configuration (Fig. 4B). The HPLC patterns of the retinal oxime isomers in the dark- and light-adapted states predominantly exhibited the peaks of all-*trans* isomers. The ratios of all-*trans* isomers were estimated to be 95 and 99%, respectively, in the dark- and light-adapted states by calculating the area under the peaks considering the molecular coefficient of each isomer as previously described^14,45^. This indicates that *Om*R2 possesses all-*trans* retinal regardless of the light environment and functions with all-*trans* retinal. It is generally known that archaeal proton pump rhodopsins, such as BR and AR3, possess both all-*trans* and 13-*cis* retinals whose ratio is dynamically changed according to the light environment^46,47^. On the other hand, bacterial and eukaryotic proton pump rhodopsins, such as PR, TR, LR and ARII, possess all-*trans* retinal predominantly both in dark- and in light-adapted conditions^9^. Similarly, *Om*R2 was found to possess all-*trans* retinal predominantly regardless of the light environment (Table 1).

**Table 1:** Molecular properties of *Om*R2 and comparison with other microbial rhodopsins (N.D., not determined).

Opsin	Origin	Absorption maximum (nm)	Retinal composition (%)	p*K*_a_ of the counterion	M-decay rate (ms^−1^)	O-decay rate (ms^−1^)	Refs.
*Om*R2	Eukarya	533	All-*trans* (95)	3.0 (Asp88)	0.16	0.03	This study
*Om*R1	Eukarya	520	All-*trans* (90)	4 (Asp100)	0.24	0.03	^37^
LR	Eukarya	542	All-*trans* (97)	N.D	0.12	0.05	^24^
ARII	Eukarya	534	All-*trans* (96)	2.6 (Asp81)	0.13	0.13	^48^
BR	Archaea	568	All-*trans* (47) 13-*cis* (53)	2.6 (Asp85)	0.3	0.1	^49,50^
AR3	Archaea	552	All-*trans* (53) 13-*cis* (47)	3.1 (Asp95)	0.45	0.03	^51^
PR	Bacteria	525	All-*trans* (95)	7.5 (Asp97)	4.0	2.8 × 10^–3^	^52^
TR	Bacteria	530	All-*trans* (98)	3.4 (Asp95)	3.85 × 10^–3^	3.95 × 10^–3^	^9^

**Figure 5:**
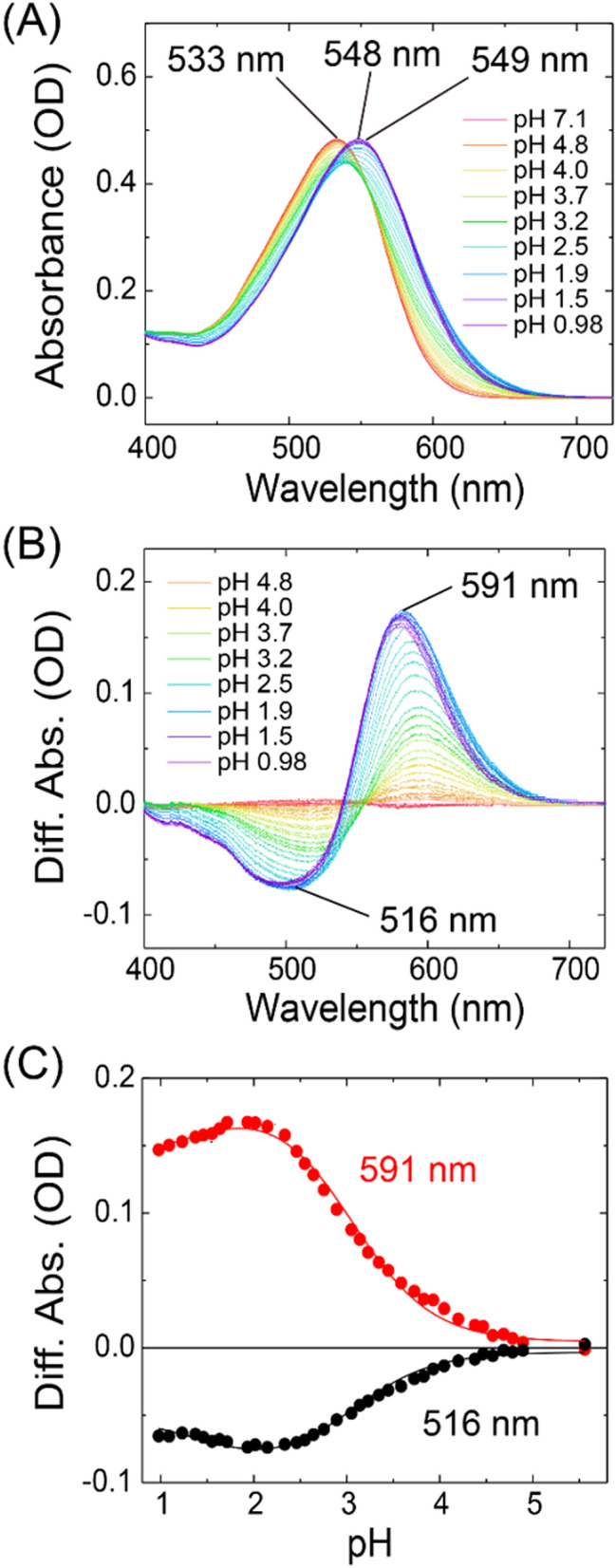
pH titration experiments of *Om*R2 at acidic conditions. (**A**) Absorption spectra of *Om*R2 at acidic pH from 7.1 to 0.98 in Buffer A containing 50 mM Tris–HCl, 1 M NaCl and 0.05% (w/v) DDM. (**B**) Difference absorption spectra; each spectrum was obtained by subtracting the spectrum at pH 7.1. (**C**) Plots of the difference absorbance at 516 and 591 nm against the pH values. The titration curve was analyzed using the Henderson–Hasselbalch equation assuming double p*K*_a_ values (solid lines).

The proton pump rhodopsins are known to transfer the substrate protons through some proton acceptable charged residues such as Asp and Glu inside the proteins via the Grotthuss mechanism^53^. The protonated Schiff base of the retinal chromophore is stabilized by an aspartic acid as a counterion, which accepts the substrate proton from the Schiff base during the photocycle for the proton pumping function. To estimate the p*K*_a_ value of the counterion of *Om*R2, we measured its spectral changes upon acidification. When the pH values decreased from 7.1 to 1.7, a large spectral red-shift from 533 to 549 nm was observed (Fig. 5A). Interestingly, when the pH value further decreased to 0.98, *Om*R2 showed a small spectral blue-shift from 549 to 548 nm. The spectral red-shift can be interpreted as the protonation of the counterion leading to a decrease in the energy gap between the electronic ground and excited state, which is commonly observed in outward proton pump rhodopsins^9,54^. Judging from the sequence alignment between *Om*R2 and other proton pump rhodopsins, Asp88 (Asp 85 in BR) is assigned to the putative counterion in *Om*R2 (Fig. 1B and Fig. S1). Since a spectral blue-shift is observed at low pH in BR and is assigned to the protonation of Asp212 as a secondary counterion^55^, the observed small spectral blue-shift at the extremely low pH would be the protonation of Asp209 (corresponding to Asp212 in BR) as a putative secondary counterion in *Om*R2 (Fig. 1B). The difference spectra showed the increase in absorbance at 591 nm and the concomitant decrease in absorbance at 516 nm. These difference spectra did not show an isosbestic point (Fig. 5B), indicating that the process of spectral changes reflects the transition between more than three states, probably the deprotonated and protonated states of the putative counterions (Asp88 and Asp209). The plots of the difference absorbance at 591 and 516 nm against the acidic pH values (Fig. 5C) were well fitted using the Henderson–Hasselbalch equation assuming two p*K*_a_ values. From the fitting analysis, the p*K*_a_ values for the spectral red-shift and blue-shift were estimated to be 3.0 ± 0.04 and 1.5 ± 0.22, respectively. Since both spectral shifts correspond to the protonation process of Asp88 and Asp209, we estimated the p*K*_a_ of the putative counterion Asp88 in *Om*R2 as 3.0 ± 0.04, and the value of the secondary putative counterion Asp209 as 1.5 ± 0.22. The p*K*_a_ of the counterion in ARII was estimated as 2.6, which is a value similar to that of *Om*R2 (Table 1). To determine the counterion residues of *Om*R2 and further clarify this issue, mutational analysis of Asp88 and Asp209 will be required as future work.

Although we attempted to estimate the p*K*_a_ of the protonated Schiff base of *Om*R2 by measuring its spectral changes upon alkalinization, the protein denaturation under alkaline conditions (> ~ pH 10.5) made the estimation impossible.

**Figure 6:**
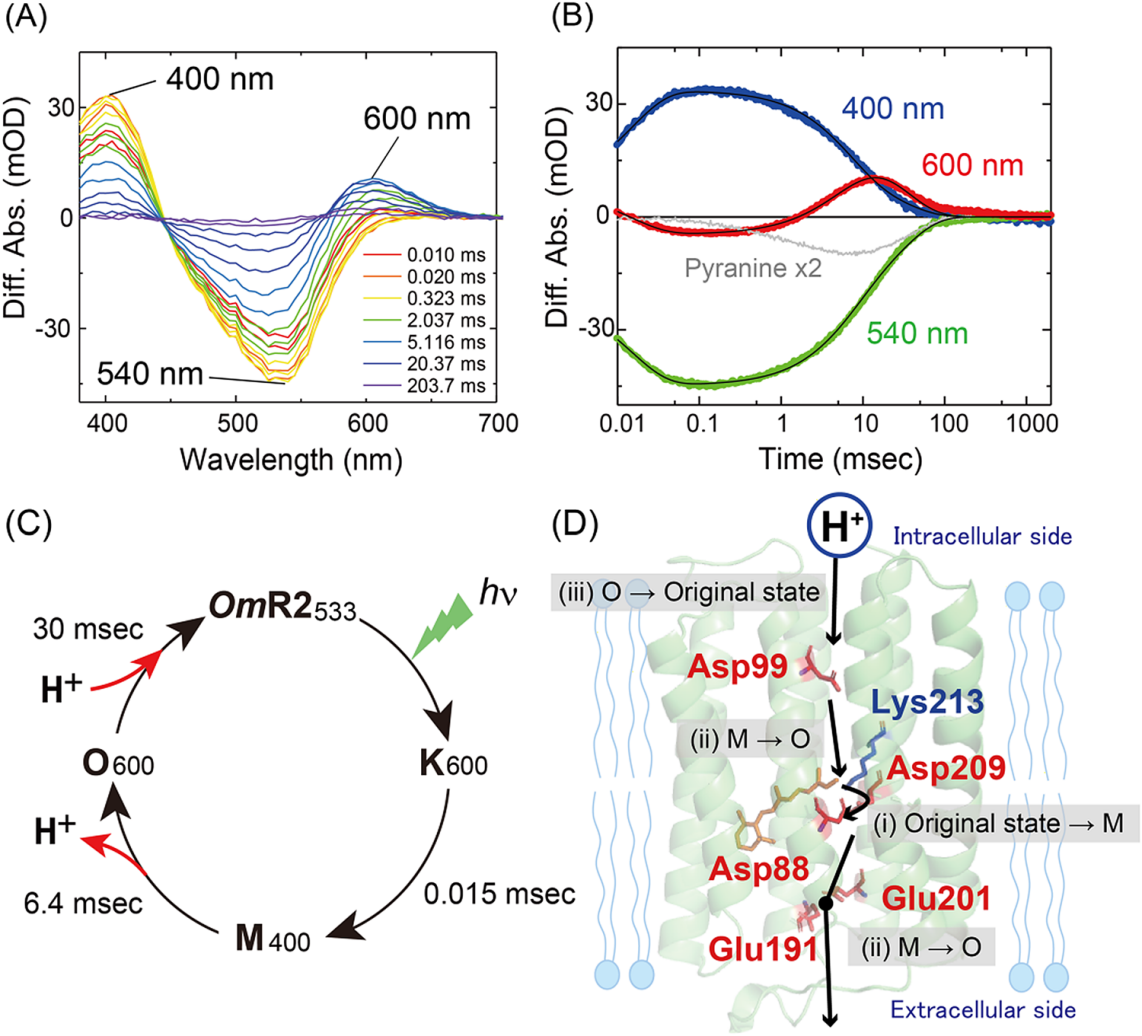
Photocycle and proton transport model of *Om*R2. (**A**) Flash-induced difference absorption spectra over the spectral range of 380 to 710 nm in Buffer A containing 50 mM Tris–HCl (pH 7.0), 1 M NaCl and 0.05% (w/v) DDM. (**B**) Time courses of absorbance changes at 400, 540 and 600 nm. The black solid lines indicate the fitting curves. The absorption changes of pyranine monitored at 450 nm were enlarged 2 times and are shown as a gray solid line. (**C**) Proposed photocycle model of *Om*R2 with the timing of the proton release and uptake. (**D**) The proton transport mechanism through some residues in *Om*R2. The pathway for putative proton transport and key residues are indicated on the homology model of *Om*R2, which was constructed from the crystal structure of ARII (PDB 3AM6) by SWISS model (https://swissmodel.expasy.org/).

To analyze the photocycle of *Om*R2, we then performed flash-photolysis experiments. Figure 6A shows the flash-induced difference spectra over the spectral range of 380–710 nm. The depletion and recovery of absorbance at ~ 540 nm correspond to the bleaching of the original state, while an increase and decrease of absorbance at ~ 400 and 600 nm were characteristically observed. Figure 6B shows the time courses of the difference absorbance changes at the three wavelengths of 400, 540 and 600 nm. Following the illumination, an absorption increase at ~ 600 nm was observed together with the depletion of the original state. An absorption increase at ~ 400 nm was then observed with a concomitant absorption decrease at ~ 600 nm within 0.1 ms. Considering the temporal and spectral ranges of the absorption changes, the absorbances at 600 and 400 nm were tentatively attributed to the K- and M-intermediates, respectively. The absorbance at ~ 400 nm decreased with the concomitant absorbance increase at ~ 600 nm, which was tentatively assigned as the O-intermediate, within 50 ms. Finally, the absorbance at ~ 600 nm was depleted with recovery of the original state within 1 s. Thus, after the light absorption, *Om*R2 sequentially forms K-, M- and O-intermediates, and then returns to the original state. To estimate the decay time constants of the intermediates, the temporal absorption changes at 400, 540 and 600 nm were fitted with a triple-exponential function assuming the irreversible sequential model. The decay time constants of the K-, M- and O-intermediates were estimated as 0.015, 6.4 and 30 ms, respectively. Finally, we investigated how proton uptake and release happen during the photocycle since *Om*R2 exhibits a proton pumping function. We measured the flash-induced absorption change at 450 nm of a pH-sensitive fluorochrome, pyranine, which reflects the solvent pH changes as previously described^14,42^. As a result, the absorbance of pyranine decreased within 10 ms and then increased within 100 ms. The time constants of the absorbance decrease and increase processes were estimated as 1.5 and 47 ms, respectively, which were consistent with the formation and decay time constants of the O-intermediate (Fig. 6B). This suggests that the substrate proton was first released from *Om*R2 upon the O-formation and then taken up from the bulk solution upon the O-decay during the photocycle.

Based on the above results, we propose a photocycle model of *Om*R2 as shown schematically in Fig. 6C. It is generally known that proton pump rhodopsins carry one substrate proton per one photocycle. That means that the strength of proton pumping activities would be proportional to the period of the photocycle, which is predominantly determined by the decay time of the late intermediate such as the O-intermediate. As the decay rate of the O-intermediate of *Om*R2 was relatively fast (0.03 ms^−1^) compared with AR3 and LR (0.03 and 0.05 ms^−1^, respectively) (Table 1), *Om*R2 can be thought to show an efficient proton pumping activity, which was demonstrated by the electrophysiological analysis (Fig. 2). As future work to clarify the photocycle model of *Om*R2, structural investigations of each photointermediate, such as vibrational spectroscopic analysis and X-ray crystallographic analysis, will be necessary.

### Comparison of the photochemical properties of *Om*R2 with other proton pump rhodopsins

The photochemical properties of *Om*R2 are listed with the well-characterized proton pump rhodopsins, *Om*R1, LR and ARII as eukaryotic rhodopsins, BR and AR3 as archaeal rhodopsins, and PR and TR as bacterial rhodopsins (Table 1). *Om*R2 possesses a deprotonated counterion (presumably Asp88 with the p*K*_a_ value of 3.0) of the protonated Schiff base (Lys213) in the unphotolyzed state and shows an absorption maximum at 533 nm with the all-*trans* retinal chromophore. These properties are similar to those of *Om*R1, LR and ARII. During the photocycle, *Om*R2 sequentially forms the red-shifted K-intermediate, the blue-shifted M-intermediate and the red-shifted O-intermediate, whose decay rates are similar to those of *Om*R1, LR and ARII. The above comparisons suggest that *Om*R2 exhibits the typical molecular and photochemical properties present in eukaryotic proton pump rhodopsins.

*Om*R2 contains the acidic amino acid residues that are key residues for function in outward proton pump rhodopsins (Fig. 1B and Fig. S1). Asp88 corresponds to the counterion (Asp85 in BR) that works as a proton acceptor from the Schiff base. Additionally, Asp99 corresponds to a proton donor (Asp96 in BR) and Glu191 and Glu201 correspond to the proton releasing group (Glu194 and Glu204 in BR). From the analogy with BR^3^, we propose the proton movement during the photocycle in *Om*R2 as follows: (i) the proton of the Schiff base is transferred to the counterion Asp88 during M-formation, (ii) the proton is released from the counterion to the extracellular side through the putative proton releasing group (Glu191 and Glu201), and simultaneously the proton of Asp99 is transferred to the Schiff base during M-decay, and (iii) the proton is taken up from the intracellular side to Asp99 during O-decay (Fig. 6D). Noteworthy, our results indicate that the proton release and uptake process correspond to the formation and decay of the O-intermediate, suggesting the structural importance of the O-intermediate of *Om*R2. The proton uptake from the intracellular side to the proton donor residue (Asp96 in BR) is generally thought to disrupt the hydrogen-bonding network between the proton donor residue and the protonated Schiff base that triggers reisomerization of retinal from the 13-*cis* to the all-*trans* form^56^. In fact, a proton is taken up during O-formation in BR and ARII^3,48^. In contrast, a proton is taken up during “O-decay” in *Om*R2, suggesting its structural difference of the O-intermediate. The structural features of the O-intermediate should be analyzed by structural and vibrational spectroscopic measurements to further understand of the detailed proton pumping mechanism in the future. By taking advantage of the successful expression of *Om*R2 in *E. coli* cells, *Om*R2 will be a good model to analyze the functional mechanism of eukaryotic rhodopsins using structural and spectroscopic measurements in the future.

## Methods

### Construction of the phylogenetic tree of microbial rhodopsins

The protein sequences of eukaryotic rhodopsins, which were previously reported as putative proton pump rhodopsins, and rhodopsins from *O. marina* were obtained from the Genbank database. The protein sequences for *Oxyrrhis marina* rhodopsin-1 (*Om*R1, Genbank accession number; ABV22426), *Oxyrrhis marina* rhodopsin-2 (*Om*R2, AIN36546), *Oxyrrhis marina* rhodopsins (ADY17806, ADY17809, ABV22427, ABV22430, ABV22432, AIN36547, AIN36548, AIN36549) (Fig. 1B), *Leptosphaeria maculans* rhodopsin (LR, AAG01180)^24^, *Phaeosphaeria nodorum* rhodopsin-1 (SNOG_00807)^57^, *Phaeosphaeria nodorum* rhodopsin-2 (SNOG_00341)^57^, *Bipolaris oryzae* rhodopsin-1 (AB489199)^58^, *Bipolaris oryzae* rhodopsin-2 (AB489200)^58^, *Sclerotinia sclerotiorum* rhodopsin-1 (XP_001597420)^59^, *Sclerotinia sclerotiorum* rhodopsin-2 (XP_001594532)^59^, *Botrytis cinerea* rhodopsin (BC1G_02456)^60^, *Aureobasidium pullulans* rhodopsin (KEQ87154)^61^, *Acetabularia acetabulum* rhodopsin I (ARI, AEF12206)^62^, *Acetabularia acetabulum* rhodopsin II (ARII, AEF12207)^48^, *Chlorella vulgaris* rhodopsin (JQ255360)^21^, *Coccomyxa subellipsoidea* rhodopsin (CsR, XP_005646688)^63^, *Neurospora crassa* rhodopsin (NR, AAD45253)^17^, *Fusarium fujikuroi *rhodopsin (OpsA, CAR82401)^64^, *Fusarium fujikuroi* rhodopsin (CarO, CAD97459)^64^, *Pseudo-nitzschia granii* rhodopsin (AJA37445)^65^, *Prorocentrum donghaiense* rhodopsin (KM282617)^66^, *Pyrocystis lunula* rhodopsin (AF508258)^67^, and *Cyanophora paradoxa* rhodopsin (ACV05065)^68^ were aligned using the MUSCLE algorithm in MEGA-X software (https://www.megasoftware.net/). The phylogenetic tree was inferred using the maximum likelihood method of MEGA-X software. At this time, the substitution model was selected as the LG model, a discrete Gamma distribution was used to model evolutionary rate differences among sites (five categories, + G parameter = 2.32), and the rate variation model allowed for sites to be evolutionarily invariable (1.94% sites). The bootstrap values were given by 100 iterations of the bootstrap test.

### Gene preparation, protein expression and electrophysiological studies of ND7/23 cells

The full-length cDNA for *Om*R2, whose codons were optimized for human codon usage, was chemically synthesized by GenScript (Tokyo, Japan) (a kind gift from Drs Haruhiko Bito and Masayuki Sakamoto). The *Om*R2 gene was inserted into the CMV promoter-based mammalian expression vector (a kind gift from Drs Hiromu Yawo and Toru Ishizuka) as previously described^39,40^. In short, enhanced yellow fluorescent protein (EYFP) was fused to the C-terminus of *Om*R2 as a reporter. Also, EYFP was flanked with a membrane trafficking signal (TS) at the N-terminus and an endoplasmic reticulum export signal (ER) at the C-terminus to improve its expression and plasma membrane localization. The TS and ER signals were “KSRITSEGEYIPLDQIDINV” and “FCYENEV”, respectively, derived from the Kir2.1 potassium channel^69^. Furthermore, the WPRE (Woodchuck hepatitis virus Post-transcriptional Regulatory Element) sequence was inserted to stabilize the transcribed mRNA and increase the amount of translated protein. The expression vector encoding *Om*R2 was prepared with an In-Fusion cloning Kit (Takara Bio, Japan) according to the manufacturer’s instructions as previously described^28,70^.

Electrophysiological measurements were carried out at room temperature (20–25 °C) with ND7/23 cells. ND7/23 cells were cultured in Dulbecco’s Modified Eagle Medium (Gibco, DMEM/F12, Thermo Fisher Scientific Life Sciences, USA) supplemented with 10% fetal bovine serum, 0.0625% (w/v) penicillin and 0.01% (w/v) streptomycin under a 5% CO_2_ atmosphere at 37 °C. The expression plasmid was transiently transfected into cells using the calcium-phosphate method^45^. After 6 h incubation of the transfected cells, all-*trans* retinal (final concentration = 1 µM) was added into the medium. Electrophysiological experiments were conducted 48–60 h after transfection. Transfected cells were identified by the presence of EYFP fluorescence. The fluorescence signals for EYFP were observed using an IX71 inverted microscope (Olympus, Japan) with a fluorescence mirror unit (U-MYFPHQ, Olympus) and a mercury lamp (U-LH100HGAPO, Olympus). Photocurrents were measured using an EPC 10 USB computer-controlled Patch Clamp Amplifier (HEKA Elektronik, Germany) under a whole-cell patch clamp configuration. The data were analyzed with Patch master software (HEKA Elektronik, Germany). The internal pipette solution for whole-cell voltage clamp recordings from ND7/23 cells contained 50 mM HEPES, 140 mM CsCl, 3 mM MgCl_2_, 5 mM Na_2_EGTA and 2.5 mM MgATP, adjusted to pH 7.3 with CsOH. The cells were continuously superfused by an extracellular medium (10 mM HEPES, 138 mM NaCl, 3 mM KCl, 1 mM MgCl_2_, 2 mM CaCl_2_, 0.1 M glucose, adjusted to pH 9.0, 7.4 and 5.0 with NaOH or HCl). To investigate pump activity in the absence of extracellular chloride, sodium and the presence of potassium, NaCl was also substituted by the same amount of sodium gluconate, CsCl, KCl. Current traces were recorded at − 60, − 40, − 20, 0, 20, 40 and 60 mV. The cells were illuminated with a white LED (THORLABS, USA) through a band-pass filter (520 ± 10 nm), where the light intensity was adjusted to 0.98 mW mm^−2^.

### Gene preparation, protein expression and purification of HEK293T cells

For protein expression and purification, the human codon-optimized *Om*R2 gene was inserted into a CAG promoter-based mammalian expression vector, pCAGGS, as previously described^29^. A hexa-histidine tag was fused to the C-terminus of *Om*R2. The expression vector encoding *Om*R2 was prepared using an In-Fusion cloning Kit. For protein expression in HEK293T cells, the plasmid was transfected into the cells using the calcium-phosphate method^29,38^. After 1 day incubation, all-*trans*-retinal (final concentration = 5 µM) was added to the transfected cells^29,38^. After another day of incubation, the cells were collected by centrifugation and were then solubilized with 1.0% (w/v) n-dodecyl-β-d-maltoside (DDM, DOJINDO Laboratories, Japan). The solubilized fraction was purified by Ni^2+^ affinity column chromatography with a linear gradient of imidazole as described previously^29^. The purified protein was concentrated by centrifugation using an Amicon Ultra filter (30,000 M_w_ cut-off; Millipore, USA). The sample was then loaded into and eluted from a PD-10 column (GE-Healthcare, UK) with Buffer A (50 mM Tris–HCl, pH 7.0, 1 M NaCl and 0.05% (w/v) DDM).

### Gene preparation, protein expression and ion transport measurements of *E. coli* cells

The full-length cDNA for *Om*R2, whose codons were optimized for *E. coli* codon usage, were chemically synthesized by Eurofins Genomics and inserted into the NdeI-XhoI site of the pET21a(+) vector as previously described^14^. A hexa-histidine-tag was fused at the C-terminus of *Om*R2, which was utilized for purification of the expressed protein. The procedures for protein expression were essentially the same as previously described^14,42^. *E. coli* BL21(DE3) cells harboring the cognate plasmid were grown at 37 °C in LB medium supplemented with ampicillin (final concentration = 50 µg mL^−1^). Protein expression was induced at an optical density at 600 nm (OD_600_) of 0.8–1.2 with 1 mM isopropyl β-d-1-thiogalactopyranoside (IPTG) and 10 μM all-*trans* retinal, after which the cells were incubated at 37 °C for 3 h. The proton transport activity of *Om*R2 was measured as light-induced pH changes of suspensions of *E. coli* cells as previously described^14,42^. In short, cells expressing *Om*R2 were washed more than three times in 150 mM NaCl and were then resuspended in the same solution for measurements. Each cell suspension was placed in the dark for several min and then illuminated using a 300 W Xenon lamp (ca. 30 mW cm^−2^, MAX-303, Asahi spectra, Japan) through a > 460 nm long-pass filter (Y48, HOYA, Japan) for 3 min. Measurements were repeated under the same conditions after addition of the protonophore carbonyl cyanide m-chlorophenylhydrazone (CCCP) (final concentration = 10 μM). Light-induced pH changes were monitored using a Horiba F-72 pH meter. All measurements were conducted at 25 °C using a thermostat (Eyela NCB-1200, Tokyo Rikakikai Co. Ltd, Japan).

### Purification of *Om*R2 from *E. coli* cells and spectroscopic measurements of the purified protein

*Escherichia coli* cells expressing *Om*R2 were disrupted by sonication for 30 min in ice-cold water in Buffer B containing 50 mM Tris–HCl (pH 7.0) and 300 mM NaCl. The crude membrane fraction was collected by ultracentrifugation and solubilized with 1.0% (w/v) DDM. The solubilized fraction was purified by Ni^2+^ affinity column chromatography with a linear gradient of imidazole as described previously^14,42^. The purified protein was concentrated by centrifugation using an Amicon Ultra filter (30,000 M_w_ cut-off; Millipore, USA). The sample media was then replaced with Buffer A by ultrafiltration for 3-times.

Absorption spectra of purified proteins were recorded using a UV-2450 spectrophotometer (Shimadzu, Japan) at room temperature in Buffer A. The retinal composition in *Om*R2 was analyzed by high-performance liquid chromatography (HPLC) as described previously^14^. For light-adaptation, the samples were illuminated for 3 min at 530 ± 10 nm, where the light power was adjusted to ~ 10 mW cm^−2^. The molar compositions of the retinal isomers were calculated from the areas of the peaks in HPLC patterns monitored at 360 nm using the extinction coefficients of retinal oxime isomers as described previously^14,45^. For pH titration experiments, the samples were suspended in Buffer A. The pH values of the samples were adjusted to the desired acidic values by adding HCl, after which the absorption spectra were measured at each pH value. All measurements were conducted at room temperature (approx. 25 °C) under room light. After the measurements, the reversibility of the spectral changes was checked to confirm that the sample was not denatured during the measurements. The absorption changes at specific wavelengths were plotted against pH values and the plots were fitted to the Henderson–Hasselbalch equation assuming double p*K*_a_ values as previously described^14^.

Transient time-resolved absorption spectra of the purified proteins from 380 to 700 nm at 5 nm intervals were obtained using a homemade computer-controlled flash photolysis system equipped with an Nd:YAG laser as an actinic light source^14,42^. By using an optical parametric oscillator, the wavelength of the actinic pulse was tuned at 530 nm for *Om*R2. The pulse intensity was adjusted to 2 mJ per pulse. All data were averaged to improve the signal-to-noise ratio (n = 30). All measurements were conducted at 25 °C. For these experiments, the samples were suspended in Buffer A. After the measurements, the reproducibility of the data was checked to confirm that the sample was not denatured during the measurements. To investigate proton uptake and release during the photocycle, we used the pH indicator pyranine (final concentration = 100 µM, Tokyo Chemical Industry Co., Ltd, Japan), which has been extensively used to monitor light-induced pH changes in various rhodopsins^14,42^. The pH changes in the bulk environment were measured as the absorption changes of pyranine at 450 nm. The absorption changes of pyranine were obtained by subtracting the absorption changes of samples without pyranine from those of samples with pyranine. The experiments using pyranine were performed in an unbuffered solution containing 1 M NaCl and 0.05% (w/v) DDM (pH 7.0) to enhance the signals. The results of 1000-traces were averaged to improve the signal-to-noise ratio.

## Supplementary Information

Supplementary Figure S1.
