# Supplementary material for: Functional expression of the eukaryotic proton pump rhodopsin OmR2 in Escherichia coli and its photochemical characterization
Source: Sci Rep. 2021 Jul 20;11:14765. doi: 10.1038/s41598-021-94181-w (PMC8292405; doi:10.1038/s41598-021-94181-w)
Supplement: Supplementary file 1 — Supplementary Figure S1. [file 41598_2021_94181_MOESM1_ESM.docx]

**Supplementary information**

**Functional Expression of the Eukaryotic Proton Pump Rhodopsin OmR2 in Escherichia coli and Its Photochemical Characterization**

Masuzu Kikuchi^1, #^, Keiichi Kojima^1,2, #^, Shin Nakao^1^, Susumu Yoshizawa^3^, Shiho Kawanishi^2^, Atsushi Shibukawa^2^, Takashi Kikukawa^4,5^ & Yuki Sudo^1,2,*^

^1^Division of Pharmaceutical Sciences, Okayama University, Okayama 700-8530, Japan.

^2^Graduate School of Medicine, Dentistry and Pharmaceutical Sciences, Okayama University, Okayama 700-8530, Japan.

^3^Atmosphere and Ocean Research Institute, The University of Tokyo, Chiba 277-8564, Japan.

^4^Faculty of Advanced Life Science, Hokkaido University, Sapporo 060-0810, Japan

^5^Global Station for Soft Matter, GI-CoRE, Hokkaido University, Sapporo 001-0021, Japan

^#^These authors contributed equally to this work

*To whom correspondence should be addressed.

Yuki Sudo; Telephone: +81-86-251-7945, E-mail: sudo@okayama-u.ac.jp


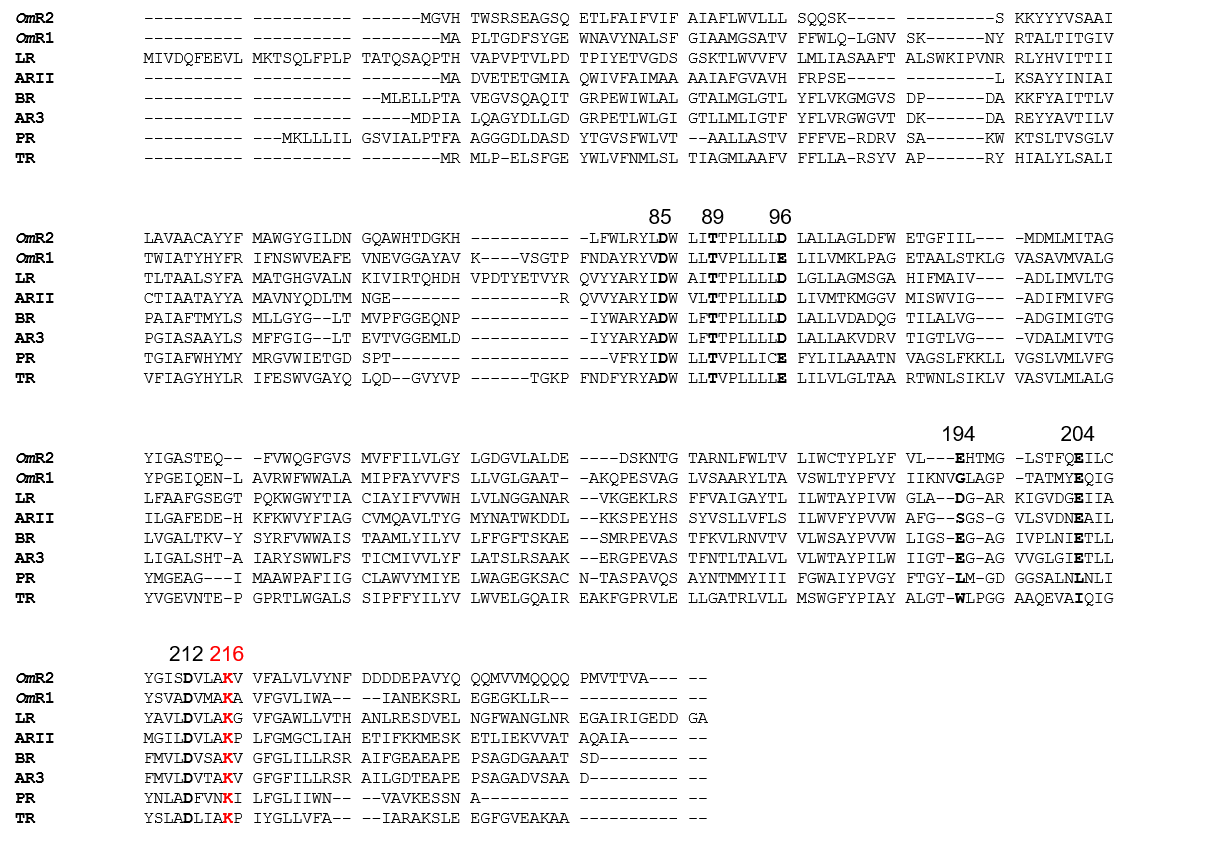


**Figure S1. Amino acid sequence alignment of the well-characterized proton pump rhodopsins with *Om*R2.** Amino acid sequence alignment of proton pump rhodopsins, *Om*R2 (AIN36546), *Om*R1 (ABV22426), LR (AAG01180), ARII (AEF12207), BR (P02945), AR3 (P96787), PR (Q9F7P4) and TR (WP_014629850). The numbers of amino acids in BR are indicated above the columns. The known functions of the amino acids are as follows: primary proton acceptor (Asp85), proton donor (Glu96), proton release group (Glu194 and Glu204), counterion (Asp212), and the Schiff base (Lys216). The Schiff base Lys (K) is shown in red.
